# Prophylactic HPV vaccines: prospects for eliminating ano-genital cancer

**DOI:** 10.1038/sj.bjc.6603695

**Published:** 2007-03-20

**Authors:** M Stanley

**Affiliations:** 1 Department of Pathology, University of Cambridge, Tennis Court Road, Cambridge CB2 1QP, UK

**Keywords:** vaccines, HPV16/18 L1 VLPs, neutralising antibody, cervical cancer, CIN2/3, protection

## Abstract

Virtually all cases of cervical cancer and its precursor intra-epithelial lesions are a result of infection with one or other of a subset of genital human papillomaviruses (HPVs), suggesting that prevention of HPV infection by prophylactic vaccination would be a highly effective anticancer strategy. Two HPV L1 virus-like particle vaccines have been developed, a quadrivalent HPV16/18/6/11 product and a bivalent HPV16/18 product; both have been shown to be highly immunogenic with a good safety profile and 100% efficacy against HPV16/18-related high-grade cervical intra-epithelial neoplasia (CIN2/3), implying that they will be effective at preventing HPV16/18-related cervical cancer.

Papillomaviruses are a large family of small, double-stranded DNA viruses that cause benign epithelial proliferations or warts in the natural infection. These viruses have two key characteristics; first they are absolutely host-specific, thus rabbit viruses only infect rabbits, dog viruses only infect dogs, human papillomaviruses (HPVs) only infect humans, secondly they are tissue-specific, a complete infectious cycle occurs only in a fully differentiating keratinised squamous epithelium. The HPVs are a very large family, classified by genotype (DNA sequence) not by serotype. To date, at least 200 HPVs have been isolated from tissue biopsies and, of these, approximately 100 have been fully sequenced. Despite their very large numbers, HPVs can be classified into two large groups: those that infect skin, or cutaneous surfaces, and those that infect the internal wet-squamous mucosal surfaces. Within these groups there are low-risk types, which generate benign lesions, in other words, warts, and high-risk or oncogenic types, which are associated with cancers and their precursor lesions.

This is particularly apparent in the genital tract, where approximately 40 HPV types regularly or sporadically infect the mucosal epithelial surfaces. Low-risk HPV types, HPV6 and 11 cause more than 90% of genital warts with minor types (HPV42, -44) and some high-risk HPVs contributing to about 10%. Oncogenic HPVs in the genital tract are dominated by HPV16 and HPV18 which, with their close relatives, 31, 33, 35, 52, 58, 39, 45, 59, 56, 66 and 51, are the cause of cervical cancer (Clifford *et al*, 2003). Thus in 99% or more of biopsies of invasive cervical cancer worldwide, HPV DNA sequences can be detected (Walboomers *et al*, 1999), and in the high-risk precursor lesions, cervical intra-epithelial neoplasia (CIN2, -3) approximately 80% contain the high-risk HPVs (Clifford *et al*, 2003). HPV16 dominates, with at least 50% of cancers irrespective of geographical location, containing HPV16, followed by HPV18, 7 to 20%. Human papillomavirus infection is not only the cause of invasive cervical cancer and hrDNA sequences are found in a proportion of anal, vulvar, vaginal, penile and head and neck cancers (Table 1). HPV16 is the dominant oncogenic type followed by HPV18 and overall, the malignant burden attributable to HPV infection is calculated to be 3.71% of all cancers (Parkin and Bray, 2006)

The natural history of cervical intraepithelial neoplasia CIN is, in effect, the natural history of HPV infection in the cervix. Transient genital HPV infections, both of high- and low-risk HPVs, are very common in young sexually active women with the lifetime risk for acquiring a genital HPV infection in the UK of the order of 70 to 80%. Most of these infections are transient and resolve. If lesions develop, they are low-grade CIN1 and represent the infectious cycle of the virus and are, in fact, flat papillomas. However, a fraction of infected women (approximately 10 to 15%) develop persistent HPV infection, and fail to clear virally infected cells possibly because of the failure to develop a strong cell-mediated immune response to HPV early proteins. These persistent infections with oncogenic HPVs are at risk for progression to the high-grade precursor lesion CIN2/3 and approximately 30 to 40% of CIN2/3 then progress on to invasive carcinoma (Muñoz *et al*, 2006).

## HPV VACCINES

Prophylactic vaccines have been and continue to be the most effective strategy for controlling viral infections and papillomaviruses should be no exception to this. The antibody response to HPV in humans is a serum neutralising antibody response to the major capsid protein L1. However, neutralising antibody titres are very low; the assay systems for detection of neutralising antibody are not particularly sensitive and not all infected individuals sero-convert (Carter *et al*, 2000). The evidence is that approximately 50% of women infected with HPV16 sero-convert; 70% sero-convert to HPV6. In men sero-conversion rates for HPV16 or 18 are not clearly known, but sero-conversion against HPV6 is found only in about 40% of men (Carter *et al*, 1995). Although neutralising antibody titres are low evidence from animal infections, including some of the earliest published works from Shope, the founding father of papillomavirus research (Shope, 1937), showed very clearly that neutralising antibody was protective. Thus if rabbits were infected systemically with the cotton tail rabbit papillomavirus (CRPV) by direct injection of virus into the muscle, papillomas did not arise in the infected animals but neutralising antibody was generated and the animals were completely resistant to viral challenge by abrasion of the epithelium. This and other data suggested very strongly that generating neutralising antibody to virus capsid protein would be an effective prophylactic vaccine strategy. Neutralising antibodies are directed against the L1 capsid protein and the generation of this antibody requires the tertiary or native structure of the protein. As these viruses cannot be grown in bulk in tissue culture and viral particles particularly of the oncogenic types are sparse in lesions, the generation of native structure, or properly folded L1 protein, was challenging. The challenge was met by the demonstration that if the L1 gene was expressed via a eukaryotic vector, such as yeast (Jansen *et al*, 1995) or baculovirus (Kirnbauer *et al*, 1992), the L1 protein was produced in large amounts and self-assembled into a macromolecular structure, a virus-like particle (VLP) an empty capsid that is geometrically and antigenically almost identical to the native virion. These VLPs were shown to generate neutralising antibody in the animal models and immunised animals were protected against high-level virus challenge (Jansen *et al*, 1995; Suzich *et al*, 1995; Kirnbauer *et al*, 1996).

Two HPV L1 VLP prophylactic vaccines have been developed, these are Cervarix™, a bivalent HPV16/18 VLP vaccine from GlaxoSmithKline Biologicals (Rixensart, Belgium), and Gardasil™ also know as Silgard, a quadrivalent HPV16/18/6/11 vaccine from Merck Vaccines (Merk & Co Inc, Whitehouse Station, NJ, USA) (Table 2). Although the numbers are small, in the according-to-protocol (ATP)groups in the Phase II trial of Cervarix there was 100% efficacy against the development of HPV16/18-asscociated high-grade CIN2/3 (Harper *et al*, 2004, 2006). In the Phase III trials of the Gardasil, the vaccine was 100% effective at preventing HPV16/18 high-grade CIN2/3 and was 100% effective at preventing HPV6/11/16 or 18-related external genital disease, that is genital warts, vulval intra-epithelial neoplasia (VIN) and vaginal intra-epithelial neoplasia (VAIN) (http://www.cdc.gov/nip/acip/slides/jun06/hpv-2-barr.pdf; Villa *et al*, 2006), and this vaccine is now licensed in many countries including those of the European Union and the USA.

The assumption is that the neutralising antibody to L1 generated by VLP vaccines or, after exposure to virus in natural infections, provides protection but what is the evidence to support this? The most convincing evidence is from preclinical experiments in dogs and rabbits, in which passive transfer of purified immunoglobulin-G from hyper-immune donors immunised with CRPV L1 VLPs, or dogs after spontaneous regression of canine oral papillomavirus (COPV), completely protected the naïve rabbit or dog recipient from challenge with high viral innocula (Breitburd *et al*., 1995; Ghim *et al*, 2000). Only animals immunised with intact VLPs generated neutralizing antibody and only purified IgG from these animals protected the naïve recipients. However, the question does remain as to how serum neutralising antibody protects against exclusively intra-epithelial infections of the cervix and lower genital tract mucosa and skin. A persuasive hypothesis has been that virus entry requires microtrauma to the epithelial surface and there is some recent evidence (J Roberts, personal communication) in an experimental model of infection that this can occur on infection with epithelial denudation but with the retention of a basement membrane, a scenario that would likely result in serous exudation and rapid access of serum IgGs to the virus particles. Furthermore, the portio surface of the cervix and the upper vaginal epithelium are bathed in cervical mucous, and the dominant immunoglobulin in cervical mucous is IgG transudated across the endo-cervical and squamo-columnar surfaces (Nardelli Haefliger *et al*, 2003). So, there is clearly rapid and easy access of serum antibody to the virus particles explaining the extraordinary efficacy of the VLP vaccines.

These vaccines are clearly remarkably efficacious in the short term but what is the duration of the protection induced by the vaccines? Will we need frequent booster vaccines? There is very encouraging data from both Phase II and Phase III trials showing that serum antibody levels fall from the peak levels achieved after the third immunisation to a lower concentration that persists at the same level for at least 60-months post-vaccination (Harper *et al*, 2006; Mao *et al*, 2006; Villa *et al*, 2006). The antibody concentrations in the plateau period in vaccinees are still 10 to 20 times that of natural infection. This data, combined with the evidence from large-scale population studies of naturally-infected individuals in which it can be shown that the antibody levels persist for at least 10 years after natural infection (af Geijersstam *et al*, 1999), leads to optimism that these vaccines will induce very long-lasting protection. The immune correlates of protection have so far not been established because all individuals in the trials appear to have sero-converted and there have been no evident vaccine breakthroughs.

The humoral immunity induced appears to be predominantly type-specific and this raises the question of whether there is any cross-protection afforded by the vaccines as there is considerable amino-acid sequence homology in L1 between closely related HPV types (Chen *et al*, 2000). There is evidence from the Phase II trial of Cervarix that HPV16/18 vaccinees are partially protected against incident infection with HPV31 and HPV45 (Harper *et al*, 2006). The possibility of cross-protection from the VLP vaccines is strengthened by the evidence that cross-reactive antibodies against HPV31/45/52 and 58 are generated after vaccination with Gardasil (Smith JV, personal communication) and, importantly, cross-neutralising antibodies to HPV45 and 31 are generated after vaccination with this vaccine albeit at concentrations 1 to 2 log lower than the dominant type-specific neutralising antibodies. It must be emphasised however that, at the present time, there is no evidence for cross-protection against HPV45 or 31 induced CIN2/3 and if such cross-protection does occur, it is likely to be partial and not complete. This does imply that second generation vaccines will need to include other HPV types and a frequently asked question is, will we need different ‘cocktails’ of HPV types for different populations. This seems unlikely. HPV16 and HPV18 are the dominant types worldwide consistently detected in 70% of all cervix cancers. A further six types, HPV45/31/56/52/35 and 33, consistently make up the remaining 20 to 30%, irrespective of the geographic region and a polyvalent vaccine that contained these eight types would effectively protect against more than 90% of all cervix cancers (Clifford *et al*, 2006).

A question that is currently generating more heat than light is, who and when to vaccinate? HPV L1 VLP vaccines are prophylactic not therapeutic preventing, not treating infection, and the available evidence is clear that immunisation with them will not be effective in individuals with established HPV infections of the types included in the current vaccines. Genital HPV infection is usually, but not always, sexually transmitted and for effective population-based programmes, immunisation should precede the sexual debut and the target population for vaccination, should therefore be eleven to 12-year-old pre-pubertal girls. Immunologically this is the optimal time because the immune system ‘ages’ after puberty. Immunogenicity bridging studies from the quadrivalent vaccine looking at antibody concentrations achieved after immunisation in 9- to 15-year-old girls and boys have shown that antibody levels after HPV VLP vaccination are higher in 9- to 10-year-old girls than 13- to 15-year-old girls, and higher in 13- to 15-year-old girls than 16 to 23-year-old women. The levels are higher in 9- to 15-year-old boys than in 9- to 15-year-old girls, suggesting that male immunisation will be effective (Block *et al*, 2006). Certainly if herd immunity against HPV is to be achieved, and virus transmission interrupted effectively, then boys, as well as girls, should be immunised. However, all the efficacy trials have included women only and there is no efficacy data in men available although trials are ongoing. The arguments against vaccinating boys against the oncogenic HPVs are based on health economic considerations and cost effectiveness. In a heterosexual population, the spread of HPV infection can be stopped entirely by complete protection of one sex alone and dynamic simulation models of HPV transmission show that if high coverage of females can be achieved, there is little to be gained in the additional reduction of cervical cancer by vaccinating males.

What about older women? Will these vaccines have any efficacy in women who are sexually active and who may have been exposed to HPV? The Phase III trials have shown that vaccination of HPV16 DNA-negative women – 16- to 23-year olds – does protect against the development of HPV16/18-related CIN2 or -3, implying that women in this age group, who are HPV16/18 DNA-negative, can be vaccinated with confidence, in that they will be protected against the development of 16/18-related disease. However, it is highly unlikely that there will be prescreening for HPV infection in any group, and sexually active women who are immunised would therefore have to continue in a cervical cancer screening programme, if that is available. The future of screening is another frequently raised topic, will these vaccines eliminate the need for cervical cancer screening programmes? The answer is, absolutely not in the short or medium term. It seems clear that if the current HPV prophylactic vaccines are introduced for mass immunisation in countries with effective cervical screening programmes, then the latter will have to continue unless significant cross-protection is induced by VLP immunisation to the extent that the protection against cervix cancer afforded by the vaccines is significantly greater than the prevention of cervical cancer achieved with the best population-based screening programmes, such as those in the UK. The cervical screening programme in England, at present, covers 80 to 82% of women at risk, and prevents 90% of cervical cancers in that group, with an overall protection from cervix cancer of the order of 76%. To discontinue this cancer-screening programme would require the vaccines to achieve at least 85 to 90% prevention of cervical cancer. Even if immunisation with the current vaccines was introduced for the 11- to 12-year old cohort, both immunised and non-immunised population cohorts would have continued in a screening programme as the immunised group would still be at risk from the oncogenic types not in the vaccine. What can be predicted is, that if vaccination is introduced the nature of screening will change both in terms of screening methods, with the use of molecular markers and HPV testing rather than cytology and microscopy, and the number of occasions on which women are screened.

## HUMAN PAPILLOMAVIRUS VACCINES IN THE DEVELOPING WORLD

The results from the VLP vaccine trials are immensely encouraging and exciting but the great burden of cervix cancer is in women in the underdeveloped world and delivery of the HPV L1 vaccines to these women faces some major hurdles. Vaccines for these women must be cheap and easily delivered. The VLP vaccines will at least initially be expensive; thus the vaccine is not yet widely available but where it has been licensed the current price is over $100 per dose (with three doses required to achieve full protection) although manufacturers have declared their willingness to tier prices for countries of different economic settings. The price at which the vaccine is available is almost certainly going to be a major determinant of the cost and affordability of any vaccine programme. Universal programmes for delivering health care to preadolescents are rare in the developing world, so the costs of establishing and maintaining a new system for HPV vaccination are likely to be considerable. Furthermore, social and cultural values in many countries will make vaccination of female adolescents extremely difficult. Infant vaccination reaches more than 70% of children in the world even in the poorest countries and realistically if HPV vaccines are to be delivered in such settings then it will be as infant vaccines. However, immunogenicity-bridging trials to support this have not been implemented.

An important issue is whether the L1 vaccines will both be, and remain, efficacious in immunocompromised subjects particularly those who are HIV infected. In developing countries, there is limited access to effective antiretroviral therapy raising questions of safety, immunogenicity and the induction of effective immune memory in such individuals. Clinical trials in HIV-infected individuals are underway but no data are available as yet, although one might predict that limited protection will be achieved in individuals with low T-cell counts.

## CONCLUSIONS

The HPV L1 VLP vaccines are immensely important developments in public health and the benefits that they promise are immense, offering the opportunity to prevent, in the long term, 80% of cervical cancers, 60% of vulval and 90% of anal cancers in women. They will impact significantly in the short to medium term on the incidence of high-grade CIN reducing the number of women who have to undergo large loop excision of the transformation zone (LLETZ) and economically, in the long term, they will reduce health-care costs. But the overwhelming benefit is the major improvement for human health and well-being of women everywhere.

## Figures and Tables

**Table 1:** The burden of malignant disease attributable to HPV infection

		**Developed countries**			**Developing countries**		
**SITE**	**Attrib to HPV (%)**	**Total cancers**	**Attrib to HPV**	**% all cancer**	**Total cancers**	**Attrib to HPV**	**% all cancer**
*HPV infection – attributable cancer in 2002 (developed and developing countries)*							
Cervix	100	83 400	83 400	1.7	409 400	409 400	7.0
Penis	40	5200	2100	0.0	21 100	8400	0.1
Vulva, Vagina	40	18 300	7300	0.1	21 700	8700	0.1
Anus	90	14 500	13 100	0.3	15 900	14 300	0.2
Mouth	3	91 200	2700	0.1	183 100	5500	0.1
Oro Pharynx	12	24 400	2900	0.1	27 700	3300	0.1
All Sites		5 016 100	111 500	2.2	5 827 500	449 600	7.7

It is estimated that 70% of all cervix cancers irrespective of geographical region are owing to HPV16/18 (Parkin and Bray, 2006). The current prophylactic vaccines might be expected therefore to reduce the incidence of cervical cancer over the next several decades in unscreened populations by up to 70% depending upon vaccine coverage. In well-screened populations the first effects would be a reduction in low-grade cervical abnormalities and CIN 3. It is estimated that up to 30% of low grade disease and 50% of CIN would be prevented in the vaccinated populations.

**Table 2:** HPV L1 VLP vaccine profiles

	**Gardasil™**	**Cervarix™**
L1 VLP Antigens	HPV6 20 *μ*g	HPV16 20 *μ*g
	HPV 11 40 *μ*g	HPV 18 20 *μ*g
	HPV 16 40 *μ*g	
	HPV 18 20 *μ*g	
Expression system	Yeast (*Saccharomyces* *cerevisiae*)	Baculovirus
		
Adjuvant	Proprietary aluminum hydroxyphosphate Sulfate (225 *μ*g)	ASO4 aluminum hydroxide (500 *μ*g) plus 50 *μ*g 3-deacylated monophosphoryl lipid A
Injection volume and immunisation schedule	0.5 ml i.m. 0, 2 and 6 months	0.5 ml i.m. 0, 1 and 6 months
Adolescent safety/immunogenicity bridging trials	Females and males 9–15 years	Females 10–14 years
		Males 10–18 years (in progress)
	Licensed	License application made

i.m.=intramuscular.
